# Evaluating the Ability of Multimodal Artificial Intelligence to Identify Endodontic Instruments: A Comparative Study of ChatGPT-4o and Gemini 3 Flash

**DOI:** 10.3390/jcm15114391

**Published:** 2026-06-05

**Authors:** Samet Tosun, Emre Çulha

**Affiliations:** 1 Department of Endodontics, Faculty of Dentistry, Pamukkale University, 20160 Denizli, Türkiye; 2 Department of Endodontics, Faculty of Dentistry, Gaziantep University, 27060 Gaziantep, Türkiye

**Keywords:** Chat-GPT, Gemini, endodontic file, image recognition, multimodal AI

## Abstract

**Background/Objectives**: Multimodal large language models (LLMs) are increasingly integrated into dental diagnostics. This study evaluated the ability of ChatGPT-4o and Gemini 3 Flash to visually identify endodontic instruments and assess their explanatory plausibility regarding instrument morphology. **Methods**: Standardized images of five endodontic file systems (Reciproc R25, Reciproc Blue, WaveOne Gold, MM One Shape, and XP-endo Finisher) were submitted to both models via their free tiers. Each image was evaluated 50 times per model (total n = 500) to assess both classification accuracy and response consistency. Visual recognition performance was measured using recall, precision, and F1-score, while the plausibility of morphological explanations was evaluated using a structured 3-point scale. **Results**: Gemini 3 Flash demonstrated significantly higher recognition performance compared to ChatGPT-4o (*p* < 0.001). The overall acceptable response rate was higher for Gemini 3 Flash (94.4%, [95% CI: 91.5–97.3%]) than for ChatGPT-4o (67.2%, [95% CI: 61.4–73.0%]; *p* < 0.001). Notably, Gemini 3 Flash showed strong performance in identifying complex instrument designs, whereas ChatGPT-4o exhibited marked limitations in recognizing certain non-standard geometries. Reliability analysis indicated higher consistency for Gemini 3 Flash (*κ* = 0.86, [95% CI: 0.81–0.91]) compared to ChatGPT-4o (*κ* = 0.51, [95% CI: 0.44–0.58]). **Conclusions**: Gemini 3 Flash outperformed ChatGPT-4o in both classification accuracy and consistency in this controlled visual identification task. While these findings highlight the potential of multimodal LLMs in endodontic workflows, their current performance variability limits direct, autonomous clinical application. Further validation under clinically realistic conditions is required before such systems can be considered reliable adjunctive tools.

## 1. Introduction

Recent advances in artificial intelligence (AI) and natural language processing (NLP) have introduced new approaches to medical decision-support systems. While clinical AI has historically focused on deep learning-based radiographic analysis, the emergence of multimodal large language models (LLMs) [1], such as ChatGPT (OpenAI, San Francisco, CA, USA) and Gemini (Google DeepMind, Mountain View, CA, USA), has expanded the scope of AI applications in healthcare. These models assist clinicians in information retrieval and clinical reasoning [2,3]; however, concerns remain regarding their reliability, susceptibility to bias, and the risk of hallucinations, defined as the generation of factually incorrect yet linguistically plausible outputs [4,5,6].

In dentistry, particularly in Endodontics [7,8], clinical success relies heavily on accurate visual recognition [9] and correct identification of instruments [10]. The number of available endodontic file systems has increased substantially with the introduction of Nickel–Titanium (NiTi) alloys [11], with over 150 systems currently available [12]. These instruments differ in metallurgical properties—ranging from austenitic M-Wire to heat-treated martensitic variants such as Gold and Blue alloys [13,14]—as well as in their kinematic motions (continuous rotary vs. reciprocating).

Despite these distinctions, many file systems exhibit similar morphological characteristics, making visual differentiation challenging in routine clinical practice. This challenge becomes more pronounced following repeated sterilization, which may alter or eliminate color coding. In such cases, clinicians must rely primarily on subtle geometric and structural characteristics. Incorrect identification of an instrument may lead to inappropriate use (e.g., applying continuous rotation to a reciprocating file), potentially resulting in instrument separation, canal transportation, or other procedural complications. Evidence from surgical disciplines further indicates that instrument misidentification can increase iatrogenic risk and procedural costs, particularly under high workload conditions or among less experienced operators [15].

While previous studies have explored the ability of LLMs to interpret endodontic radiographs and clinical scenarios [6,16], research evaluating their performance in purely visual, morphology-based identification tasks remains limited. Moreover, the probabilistic and non-deterministic nature of LLMs implies that identical inputs may produce variable outputs [17], raising concerns regarding consistency and reliability in clinical applications [18]. Unlike human experts, who integrate contextual knowledge with visual assessment [19], LLMs must be systematically evaluated for both accuracy and repeatability under controlled conditions before being considered for clinical decision support [17,20].

To the best of our knowledge, no previous study has quantitatively evaluated the ability of multimodal LLMs to identify endodontic instruments based solely on visual characteristics. Therefore, the aim of this study was to assess the classification accuracy, explanatory plausibility regarding morphology, and response consistency of ChatGPT-4o and Gemini 3 Flash in identifying five representative endodontic file systems with distinct metallurgical and geometric properties. The null hypothesis was that there would be no significant difference between the two models in terms of identification accuracy and response consistency.

## 2. Materials and Methods

### 2.1. Study Design and AI Model Selection

This experimental study evaluated the visual recognition performance and the explanatory plausibility of raw outputs generated by two widely utilized multimodal LLMs: ChatGPT-4o and Gemini 3 Flash. Both models were accessed between 1 February 2026 and 20 March 2026 via their respective web-based user interfaces using default system configurations. The models were evaluated through their standard free tiers rather than API access. No manual adjustments to model settings (e.g., temperature or top_p) were performed, and all responses were generated using the default configurations provided by the platforms at the time of data collection. To simulate a standard clinical consultation and ensure out-of-the-box vision–language performance, no fine-tuning, plugins, or external tools were utilized. All evaluations were conducted via the Google Chrome browser (English-language interface) connected to European region servers. To ensure strict methodological reproducibility and prevent conversation persistence, history tracking, or backend cross-contamination, each query was initiated exclusively using newly opened “temporary chats” without memory, custom instructions, or personalized user profiles enabled. Furthermore, before initiating each independent query iteration, the browser cache, cookies, and local session histories were completely cleared to eliminate any browser-level confounders. Images were uploaded in original JPEG format without any preprocessing or modification. To address the inherent limitations of the free-tier framework and prevent automatic backend downgrades (e.g., from ChatGPT-4o to GPT-4o mini) caused by platform-imposed usage caps, data collection was strictly suspended immediately upon reaching the platform’s rate limits, thereby ensuring that all responses were generated exclusively by the primary foundation models. Finally, because these foundation models operate on non-deterministic, probabilistic backend sampling algorithms, and temperature could not be manually adjusted via the web user interface, a repeated-querying design was deliberately deployed to capture, quantify, and report this inherent backend stochasticity and response stability under controlled baseline conditions. This rigorous protocol involved 5 distinct image assets (cases), with each image subjected to 50 independent queries and upload iterations, resulting in a total dataset of 250 evaluation cycles per model.

This study did not involve human participants, animals, or patient data; therefore, institutional ethical approval was not required. A power analysis was performed using G*Power software (version 3.1.9.4, Heinrich Heine University, Düsseldorf, Germany). Assuming a medium-to-large effect size (f = 0.45), a significance level of α = 0.05, and a statistical power of 0.95, the minimum required sample size was calculated as 78 evaluations. Thus, the total pool of 500 independent queries derived from the repeated evaluation of five standardized images was deemed statistically sufficient to capture intra-model output variability within this exploratory benchmarking framework.

### 2.2. Image Dataset and Standardization

Five endodontic file systems representing distinct metallurgical properties and geometric configurations were selected [10,12]:

A. Reciproc R25 (VDW, Munich, Germany) 25/.08, 25 mm; Metallurgy: M-Wire (Austenitic); Design: S-shaped cross-section; Kinematics: Reciprocation.

B. One Shape (Micro-Mega, Besançon, France) 25/.06, 25 mm, Metallurgy: Austenitic (Electropolished), Asymmetrical cross-section, Continuous Rotation.

C. Reciproc Blue R25 (VDW, Munich, Germany), 25/.08, 25 mm; Metallurgy: Blue Heat-Treated (Martensitic); Design: S-shaped cross-section; Kinematics: Reciprocation.

D. XP-endo Finisher (FKG, La Chaux-de-Fonds, Switzerland) 25/.02, 25 mm; Metallurgy: MaxWire (Martensitic-Austenitic transition); Design: Non-tapered, adaptive geometry; Kinematics: Continuous Rotation (High speed).

E. WaveOne Gold Primary (Dentsply Maillefer, Ballaigues, Switzerland) 25/.07, 25 mm; Metallurgy: Gold Heat-Treated (Martensitic); Design: Parallelogram cross-section; Kinematics: Reciprocation.

**Figure 1:**
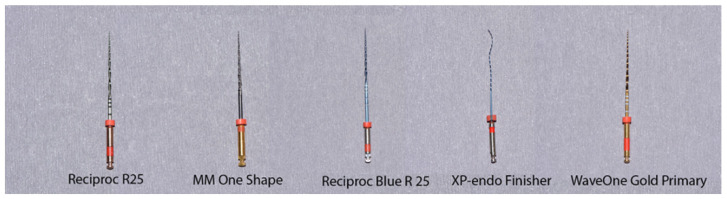
Visual catalog of the endodontic instrument systems evaluated in the study.

Images were captured using a Nikon D7200 DSLR camera (Nikon Corp., Tokyo, Japan) in JPEG format (6000 × 4000 pixels). A neutral gray background and standardized dual LED lighting were utilized to minimize external reflections and speculative color bias [21]. Camera settings were fixed (ISO 160, f/11) to ensure consistency across all images (Figure 1). Formal colorimetric calibration using a gray reference card or spectrophotometric validation was not conducted, which represents a controlled baseline condition of this visual baseline study.

### 2.3. Experimental Protocol and Prompting Strategy

A repeated-measures experimental design was employed specifically to analyze the stochastic behavior and consistency of the models. Each of the five standardized images was submitted independently to both models 50 times, yielding a total dataset of 500 distinct textual outputs (5 instruments × 2 models × 50 iterations). To maintain strict methodological boundaries and control for free-tier rate limits, data collection sessions were segmented based on platform-imposed caps, ensuring that all 250 evaluation cycles per model were executed without automatic backend model degradation.

To establish an out-of-the-box baseline utility and avoid prompting bias, a single zero-shot prompt was selected. This approach evaluates the models’ immediate visual-linguistic alignment without prompt engineering intervention. The following standardized prompt was executed:

“I will upload a photograph of an endodontic file. Please: (1) Identify the specific name of the instrument, (2) Specify its intended working motion (rotary or reciprocating), and (3) Provide a technical explanation of the visual features supporting your conclusion (e.g., color, flute geometry, taper, or cross-sectional characteristics).”

### 2.4. Scoring System and Inter-Rater Validation

Responses were independently evaluated by two experienced endodontists (each with over 10 years of clinical experience). A two-evaluator design was preferred to ensure consistency in scoring criteria across all repeated assessments. A structured 3-point grading scale was implemented to quantify the quality classification accuracy and the plausibility of the morphological explanations:

- Correct ID (2 Point): Accurate identification of the specific commercial name of the instrument coupled with a clinically plausible morphological justification.
- Partial Reasoning (1 Point): Correct identification of certain broad features (e.g., kinematics, color-coding, or taper) despite an incorrect name, or vice versa.
- Incorrect (0 Point): Complete failures in both visual identification and technical description.

Responses with a score of 1 or 2 were collectively categorized as acceptable responses. To ensure objective validation, inter-rater reliability between the two blinded evaluators was calculated using Cohen’s kappa coefficient. *κ* = 0.82, 95% CI: [0.74–0.90]).

Incorrect responses were qualitatively sub-classified into five error categories:

- Visual Misinterpretation (VM): Failure to accurately recognize or distinguish primary visual markers of the instrument.
- Reasoning Error (RE): Accurate observation of visual features but leading to an incorrect clinical or technical interpretation.
- Popularity Bias (PB): A systematic tendency of the model to misidentify a specialized instrument as a more frequently documented commercial system.
- Morphological Blindness (MB): Failure to accurately output or translate key geometric features into a correct classification, which may potentially stem from linguistic over-reliance within the multimodal fusion layer.
- Other/Technical Description (Other/TD): Generic technical responses devoid of any specific trade identification.

### 2.5. Statistical Analysis

Data analysis was performed using SPSS Statistics version 26.0 (IBM Corp., Armonk, NY, USA). Descriptive statistics were expressed as mean ± standard deviation (SD). Since the data distributions violated normality assumptions, non-parametric tests were applied. The Mann–Whitney U test was used to compare the continuous score distribution between models, and the Chi-square test was preferred for categorical comparisons. To address the potential clustering effects and pseudo-replication introduced by testing repeated queries on a fixed set of five images, the statistical analyses (including Chi-square and Mann–Whitney U tests) were strictly interpreted as exploratory assessments of response consistency, output variability, and intra-model stability under controlled baseline conditions, rather than population-level diagnostic inferences. Consequently, the use of Chi-square and Cramér’s V was explicitly framed as an exploratory measure of the strength of association between a fixed visual stimulus and the model’s categorical output stability, thereby mitigating the risk of inflated significance from quasi-independent observations. Effect sizes were reported using Cohen’s r and Cramér’s V. Fleiss’ kappa [22], along with 95% confidence intervals (CIs), was calculated to measure classification repeatability and categorical agreement across the 50 repeated iterations. Statistical significance was set a priori at *p* < 0.05.

## 3. Results

**Figure 2:**
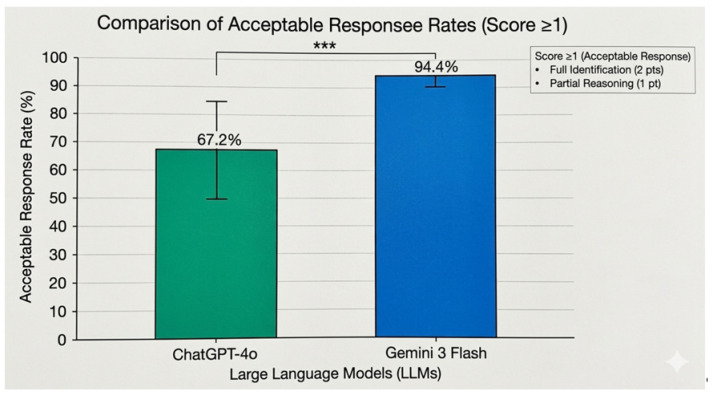
Comparative analysis of acceptable response rates (Score ≥ 1) for ChatGPT-4o and Gemini 3 Flash across the five evaluated endodontic instruments, representing the baseline visual classification reliability (*** *p* < 0.001).

A total of 500 repeated model outputs derived from five standardized instrument images (250 outputs per model) were analyzed. Gemini 3 Flash demonstrated a significantly higher visual classification performance than ChatGPT-4o, with overall correct identification rates of 69.2% [95% CI: 63.5–74.9] and 47.6% [95% CI: 41.4–53.8], respectively (*p* < 0.001). Similarly, the overall acceptable response rate (Score ≥ 1) was significantly higher for Gemini 3 Flash (94.4% [95% CI: 91.5–97.3]) compared with ChatGPT-4o (67.2% [95% CI: 61.4–73.0]; *p* < 0.001). Instrument-specific acceptable response rates are presented in Figure 2.

**Figure 3:**
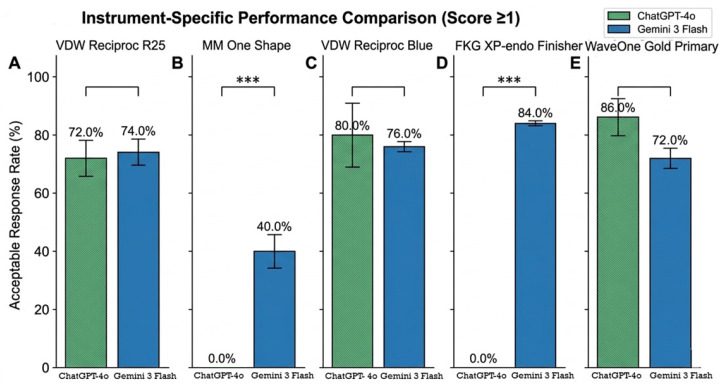
Comparative instrument-specific visual classification performance of ChatGPT-4o and Gemini 3 Flash across the five evaluated endodontic instruments. Bars represent acceptable response rates (Score ≥ 1), while numerical labels indicate correct identification percentages (*** *p* < 0.001).

Performance varied across instruments. Overall classification performance metrics, including recall, precision, and F1-score values, are summarized in Table 1. The largest difference between models was observed for the XP-endo Finisher, where Gemini 3 Flash achieved an F1-score of 0.91 [95% CI: 0.83–0.99], whereas ChatGPT-4o completely failed to correctly identify the instrument, resulting in an F1-score of 0.00 [95% CI: 0.00–0.00] (*p* < 0.001). CI values reported as [0.00–0.00] represent metrics with zero variance due to a zero true positive count. Comparative instrument-specific performance is illustrated in Figure 3.

Mean scores (0–2 scale) used to evaluate the explanatory plausibility of morphological descriptions are presented in Table 2. Gemini 3 Flash achieved significantly higher scores in several instrument categories, particularly for the XP-endo Finisher and Reciproc R25. In contrast, ChatGPT-4o demonstrated markedly lower performance in the One Shape and XP-endo Finisher instruments.

**Figure 4:**
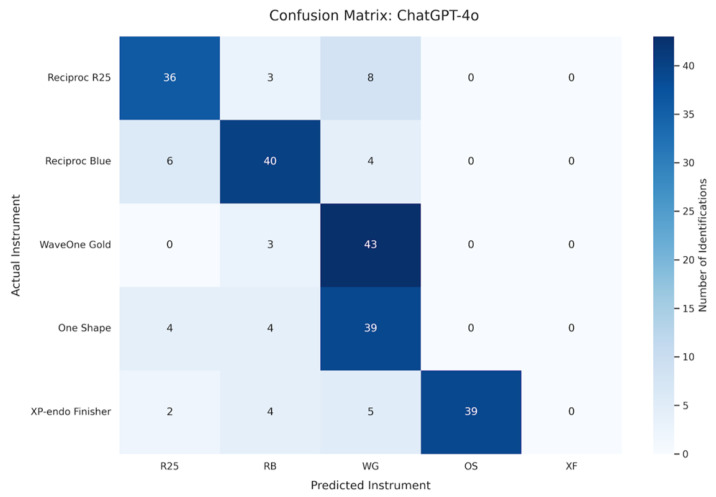
Confusion Matrix detailing the visual classification performance of ChatGPT-4o in classifying endodontic instruments. The matrix shows actual instrument types on the *y*-axis and predicted types on the *x*-axis, with values representing the number of correct and incorrect identifications per class. Notable misclassifications are observed between One Shape and WaveOne Gold, and between XP-endo Finisher and One Shape. Abbreviations: R25: Reciproc R25; RB: Reciproc Blue; WG: WaveOne Gold Primary; OS: One Shape; XF: XP-endo Finisher.

**Figure 5:**
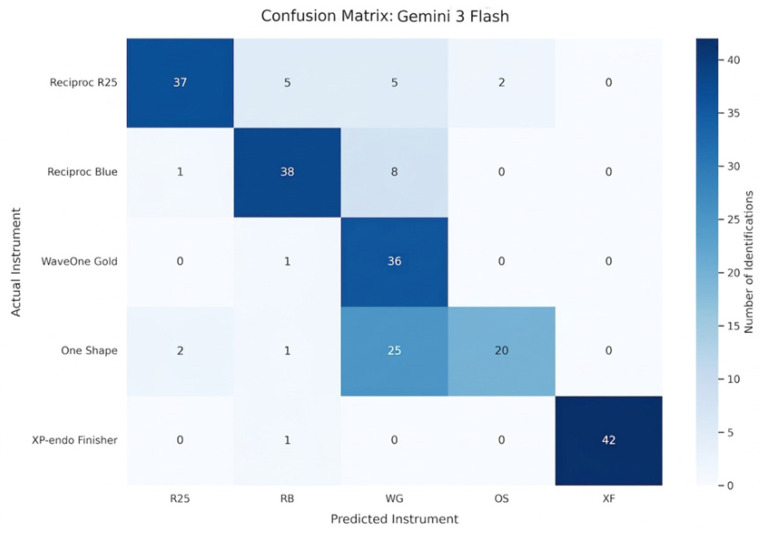
Confusion Matrix detailing the visual classification performance of Gemini 3 Flash in classifying endodontic instruments. The matrix shows actual instrument types on the *y*-axis and predicted types on the *x*-axis, with values representing the number of correct and incorrect identifications per class. The diagonal cells indicate the number of correctly identified instruments for each category. Abbreviations: R25: Reciproc R25; RB: Reciproc Blue; WG: WaveOne Gold Primary; OS: One Shape; XF: XP-endo Finisher.

Error patterns analysis revealed systematic differences between the models. ChatGPT-4o more frequently misclassified less common instruments as widely recognized systems, consistent with the predefined category of Popularity Bias. The distribution of misclassifications is shown in the confusion matrix (Figure 4). Gemini 3 Flash demonstrated fewer misclassifications for atypical instrument geometries and achieved higher identification accuracy for the XP-endo Finisher group (Figure 5).

A significant association was observed between actual instrument type and predicted classification for Gemini 3 Flash (*χ*^2^ = 563.99, *p* < 0.001). The strength of this association was large, as indicated by Cramér’s V (*V* = 0.75), indicating a strong exploratory dependency between the fixed visual stimulus and model output stability within the repeated-query controlled conditions. A significant association was also observed between actual instrument type and predicted classification for ChatGPT-4o (*χ*^2^ = 431.12, *p* < 0.001). The strength of this association was likewise large (Cramér’s V = 0.66), further substantiating the baseline response patterns of the models under fixed exploratory parameters.

Repeatability analysis demonstrated almost perfect output consistency for Gemini 3 Flash (*κ* = 0.86 [95% CI: 0.81–0.91]), whereas ChatGPT-4o showed only moderate agreement (*κ* = 0.51 [95% CI: 0.44–0.58]), indicating greater stochastic variability across repeated evaluations.

## 4. Discussion

This study evaluated the visual recognition performance of multimodal LLMs to identify endodontic instruments, demonstrating a significant disparity in execution stability. The null hypothesis was rejected, as Gemini 3 Flash showed superior classification accuracy and more consistent morphological descriptions compared to ChatGPT-4o. This variance likely reflects fundamental differences in the underlying vision–language processing strategies and token weights assigned to geometry versus color boundaries [23].

The architectural limits of these models became distinct when evaluating specialized profiles. The XP-endo Finisher, characterized by its unique zero-tapered geometry (25/.00 taper) and adaptive MaxWire alloy, presents a highly specific visual signature. Gemini 3 Flash captured with high precision (n = 42/50 F1-score: 0.91 [95% CI: 0.83–0.99]). In contrast, ChatGPT-4o completely failed to recognize these adaptive morphological markers. As clearly illustrated in the ChatGPT-4o confusion matrix (Figure 4), the model suffered from multimodal fusion failure toward the non-tapered shaft, misclassifying 39 out of 50 XP-endo Finisher images as the One Shape instrument, resulting in a true positive count of zero (Recall: 0.0% [95% CI: 0.0–0.0], Precision: 0.0% [95% CI: 0.0–0.0]).

These findings support the previous literature, which suggests that AI systems may contribute to improved diagnostic processes in healthcare settings [6,24,25]. While previous research has focused on text-based textual processing or radiographic interpretation [8,9], our results indicate that multimodal models can perform intricate visual–morphological assessments. Given the increasing diversity of NiTi systems [12], distinguishing between morphologically similar files with different heat treatments is critical for clinical safety [10]. As noted by Otsuka et al., the distinctive blue and gold colors of modern NiTi files result from specific titanium oxide layers (ranging from 60 to 140 nm) produced during thermal treatments [26]. Our findings may suggest that Gemini 3 Flash demonstrated greater sensitivity to subtle colorimetric differences between Reciproc Blue R25 and Reciproc R25, whereas ChatGPT-4o often conflated them. However, Gemini 3 Flash’s confusion matrix also revealed a bidirectional misclassification between Reciproc Blue and Reciproc R25, suggesting that while the model recognizes the S-shaped cross-section, the subtle chromatic shift from silver to blue can still pose a challenge even for advanced vision-language models.

Both models demonstrated relatively strong performance in identifying commonly used systems such as Reciproc and WaveOne Gold, which aligns with previous findings on the potential role of AI in improving clinical efficiency [2] and in medical education [27]. However, ChatGPT-4o demonstrated a tendency to misclassify less common instruments as more widely recognized systems. For example, the One Shape file, which features an asymmetrical cross-section, was frequently misidentified by ChatGPT-4o as WaveOne Gold (n = 39), a more popular reciprocating system.

The confusion matrix further reveals a systematic misclassification pattern, where less common instruments were frequently assigned to more familiar categories, supporting the presence of a potential “popularity bias,” in which models may preferentially assign unfamiliar inputs to more frequently represented categories within their training distributions. Similar frequency-dependent prediction tendencies have previously been discussed in the generative AI literature [17,28]. Similar limitations, including bias and context sensitivity, have been previously reported in medical AI applications [1,4,6]. A fundamental limitation inherent to benchmarking proprietary foundation models, which directly underscores this popularity bias, is the inability to exclude training-data contamination and benchmark leakage. Because commercial systems like the Reciproc, WaveOne, and XP-endo finisher files are heavily represented across online educational repositories, manufacturer catalogs, and the dental literature, the models possess strong prior exposure biases. Consequently, their text outputs may reflect the retrieval of highly indexed linguistic and semantic associations linked to commercial imagery, rather than an active, real-time extraction of physical geometric features from the uploaded photograph.

Crucially, these diagnostic failures should perhaps not be attributed solely to localized visual processing deficits. Rather, they may reflect a potential breakdown in the multi-modal fusion layer, where strong linguistic priors and commercial familiarity tokens within the language module could systematically override the visual tokens extracted by the vision encoder. This hypothesized semantic dominance may cause the model to confidently output a highly represented trade name even when the visual input contradicts it.

Comparing general-purpose foundation models with classical CNNs is critical for determining clinical precision [29,30]. While foundation models offer high flexibility, task-specific, narrow models trained on dental datasets provide higher accuracy and lower variance [31]. Experimental studies have demonstrated that CNN architectures are more successful than Transformer-based structures in specific clinical tasks, such as tooth structure and caries segmentation [31]. Conversely, an opposing view exists in the literature; it is argued that Vision Transformer (ViT) models outperform traditional CNNs in many dental analysis scenarios due to their ability to capture global context through self-attention mechanisms [30].

Interestingly, Gemini 3 Flash showed a higher frequency of Other/Technical Description responses, particularly for the MM One Shape file (n = 25). While this reduced its overall Correct ID score, qualitative analysis revealed that the model often provided accurate morphological descriptions (e.g., describing asymmetrical pitch or cross-section) without committing to a specific trade name. This observation may indicate a tendency toward descriptive morphological characterization rather than direct probabilistic classification, though it should be interpreted as an association of surface features rather than true visual comprehension. Both models demonstrated strong associations between instrument type and classification outcomes; however, Gemini 3 Flash showed a higher effect size (*V* = 0.75) than ChatGPT-4o (*V* = 0.66), suggesting more structured and consistent alignment between morphological features and predictive outputs.

Furthermore, the disparity in response consistency was striking. Gemini 3 Flash achieved almost perfect agreement (*κ* = 0.86, 95% CI: 0.81–0.91), whereas ChatGPT-4o showed only moderate agreement (*κ* = 0.51, 95% CI: 0.44–0.58). This highlights the non-deterministic nature of commercial LLMs’ web interfaces. Because backend parameters like temperature or top_p are locked and continuously tuned by developers, identical visual inputs can yield highly variable outputs across repeated trials [17,20]. Importantly, producing a correct response does not necessarily guarantee consistent performance across repeated trials, which may affect user trust [17]. Reliable performance is particularly critical in dentistry, where incorrect instrument identification may lead to procedural errors and increased iatrogenic risk [15,32]. Therefore, at the current stage, AI systems should be regarded as supportive tools rather than replacements for clinical expertise.

From a clinical perspective, such systems may be most useful in educational settings, preliminary instrument recognition, or decision-support workflows involving standardized imaging conditions. However, reliance on autonomous AI-based identification in complex clinical scenarios may carry risks, particularly when instruments exhibit deformation, wear, or altered surface characteristics following clinical use and sterilization.

This study has several limitations. First, only five endodontic file systems were tested; a wider variety of the 150+ available systems would provide deeper insights. Second, all evaluations were conducted using brand-new, unused instruments under optimized, standardized imaging conditions. In a true clinical environment, files undergo repeated sterilization, operational wear, micro-deformations, and surface dulling, which significantly alter their visual and colorimetric signatures. Furthermore, because only a single optimized view per instrument was provided, the models may rely heavily on gross visual priors (e.g., specific silhouette outlines or dominant metallic reflections) and memorized commercial catalog imagery (benchmark contamination) rather than executing a true, deep three-dimensional structural analysis. Third, because multiple queries were generated from a fixed image set, a substantial pseudo-replication problem and clustering effects are inherent to the data. Consequently, this study does not evaluate generalized, population-level diagnostic accuracy, but rather focuses strictly on evaluating intra-model output variability, stochastic consistency, and response stability. Although the repeated-query methodology enabled assessment of response consistency and intra-model variability, the study design was not intended to establish population-level diagnostic performance estimates. Fourth, because commercial web-based models are continuously updated without public version snapshots, exact long-term replication of these precise output ratios cannot be fully guaranteed. Model behavior and classification performance may vary across different testing periods, independent of user control. Fifth, our protocol utilized a single zero-shot prompt without chain-of-thought (CoT) prompting or few-shot examples. This was deliberately chosen to establish an out-of-the-box baseline utility, simulating how a general clinician or student would interact with these interfaces without advanced prompt engineering. However, it represents a clear limitation, as vision-language models are highly sensitive to prompt structures; incorporating iterative reasoning paths or structural templates could significantly shift their output distributions and error ratios. Sixth, a broader ethical and methodological challenge in contemporary AI research is the complete lack of transparency surrounding proprietary, closed-source architectures. Because commercial vendors do not disclose their training corpus weights, alignment filtering, or fine-tuning protocols, researchers operate within a ‘black-box’ paradigm. This opacity restricts our ability to inspect data contamination and presents long-term reproducibility limits as models undergo undocumented live updates. Finally, to mitigate the risk of pseudo-replication and clustering effects from using a fixed set of five standardized images, the statistical tests used here must be interpreted strictly as an exploratory assessment of intra-model output stability. While alternative advanced statistical frameworks, such as mixed-effects modeling or clustered bootstrapping, could provide adjusted variances for these quasi-independent observations, we retained standard non-parametric testing framed strictly as an exploratory probe into backend consistency, which remains a transparent boundary of our statistical framework.

Despite these limitations, the repeated-query design (with 50 evaluation cycles per image) represents a methodological strength by enabling evaluation of response stability beyond single-response accuracy. This is particularly relevant given the known probabilistic and non-deterministic behavior of LLM-generated outputs.

Beyond raw accuracy, operational practicality is a critical element for real-world integration. Throughout our 500-query pipeline, both platforms exhibited high uptime with zero failed image uploads or moderation refusals. However, differences in output behavior were qualitatively observed during testing sessions. ChatGPT-4o generally appeared to generate responses more rapidly but was more prone to text truncation in its qualitative descriptions, whereas Gemini 3 Flash consistently produced more complete morphological breakdowns, albeit with subjectively longer response times. Because inference latency was not formally measured, these observations should be interpreted as qualitative impressions rather than quantitative performance metrics. Finally, practical implementation of multimodal LLMs in clinical environments may depend not only on classification performance but also on computational factors such as response speed, accessibility, operational cost, and platform stability, which warrant dedicated quantitative investigation in future studies.

Future investigations should incorporate larger and more heterogeneous datasets, multiple evaluators, clinically used or deformed instruments, external validation cohorts, and more realistic imaging conditions. Integration of dynamic imaging modalities, such as video-based or three-dimensional data, may further improve the assessment of multimodal AI systems in endodontic workflows.

## 5. Conclusions

Within the controlled parameters of this benchmarking study, Gemini 3 Flash demonstrated higher visual classification accuracy and output consistency than ChatGPT-4o in identifying endodontic instruments. However, both models exhibited marked performance boundaries, particularly when processing less common geometries or specialized heat-treated alloys, where ChatGPT-4o was heavily compromised by popularity bias. Multimodal LLMs possess clear potential as adjunctive decision-support utilities and educational aids in endodontic training. However, their current probabilistic variability and susceptibility to morphological errors mean they cannot replace human clinical expertise. Future research must validate these networks against datasets containing physically degraded, worn, or deformed instruments under varied clinical imaging environments before automated classification systems can be safely deployed in practical workflows.

## Figures and Tables

**Table 1:** Comparative Visual Classification Metrics for ChatGPT-4o and Gemini 3 Flash.

Instrument Name	Model	Recall (%)	Precision (%)	F1-Score	Acceptable (Score ≥ 1)
Reciproc R25	ChatGPT-4o	72.0%	75.0%	0.73	88.0%
	Gemini 3 Flash	74.0%	92.5%	0.82	96.0%
Reciproc Blue R25	ChatGPT-4o	80.0%	74.1%	0.77	90.0%
	Gemini 3 Flash	76.0%	82.6%	0.79	94.0%
WaveOne Gold Primary	ChatGPT-4o	86.0%	43.4%	0.58	98.0%
	Gemini 3 Flash	72.0%	48.6%	0.58	92.0%
One Shape	ChatGPT-4o	0.0%	0.0%	0.00	6.0%
	Gemini 3 Flash	40.0% ***	90.9% ***	0.56 ***	90.0% ***
XP-endo Finisher	ChatGPT-4o	0.0%	0.0%	0.00	0.0%
	Gemini 3 Flash	84.0% ***	100.0%	0.91 ***	100.0% ***

Asterisks (***) indicate a statistically significant difference between ChatGPT-4o and Gemini 3 Flash within the same category (*p* < 0.001).

**Table 2:** Comparative Mean Scores and Primary Error Characterization.

Instrument Name	Model	Mean Score ± SD	Correct ID (%)	Main Error Type
Reciproc R25	Gemini 3 Flash	1.72 ± 0.45	74%	VM (Mistaken for Gold)
	ChatGPT-4o	1.60 ± 0.68	72%	VM (Silver/Gold Confusion)
Reciproc Blue R25	Gemini 3 Flash	1.68 ± 0.51	76%	VM (Blue seen as Gold)
	ChatGPT-4o	1.70 ± 0.65	80%	VM (Variant Missed)
WaveOne Gold Primary	Gemini 3 Flash	1.70 ± 0.46	80%	RE (Kinematic Mismatch)
	ChatGPT-4o	1.82 ± 0.52	86%	RE (System Mismatch)
One Shape	Gemini 3 Flash	1.30 ± 0.78 ***	40% ***	Visual Prior Bias
	ChatGPT-4o	0.06 ± 0.24	0%	Popularity Bias
XP-endo Finisher	Gemini 3 Flash	1.78 ± 0.42 ***	84% ***	RE (Shaper vs. Finisher)
	ChatGPT-4o	0.00 ± 0.00	0%	Semantic Override Bias

Asterisks (***) indicate a statistically significant difference between ChatGPT-4o and Gemini 3 Flash within the same category *p* < 0.001). VM: Visual Misinterpretation; RE: Reasoning Error.

## Data Availability

The datasets generated and analyzed during the current study are available from the corresponding author upon reasonable request.

## Abbreviations

The following abbreviations are used in this manuscript:

AI	Artificial Intelligence
DSLR	Digital Single-Lens Reflex
F1-score	Harmonic mean of precision and recall
ISO	International Organization for Standardization
JPEG	Joint Photographic Experts Group
LLM	Large Language Model
MB	Morphological Blindness
NiTi	Nickel–Titanium
NLP	Natural Language Processing
PB	Popularity Bias
RE	Reasoning Error
SD	Standard Deviation
VM	Visual Misinterpretation
*κ* (Kappa)	Fleiss’ Kappa (Inter-rater/Intra-model agreement)
